# Open versus laparoscopic liver resection for colorectal liver metastases (the Oslo-CoMet study): study protocol for a randomized controlled trial

**DOI:** 10.1186/s13063-015-0577-5

**Published:** 2015-03-04

**Authors:** Åsmund Avdem Fretland, Airazat M Kazaryan, Bjørn Atle Bjørnbeth, Kjersti Flatmark, Marit Helen Andersen, Tor Inge Tønnessen, Gudrun Maria Waaler Bjørnelv, Morten Wang Fagerland, Ronny Kristiansen, Karl Øyri, Bjørn Edwin

**Affiliations:** The Intervention Centre, Oslo University Hospital, Pb. 4950 Nydalen, 0424 Oslo, Norway; Department of HPB Surgery, Oslo University Hospital, Oslo, Norway; Institute of Clinical Medicine, University of Oslo, Oslo, Norway; Department of Surgery, Finnmark Hospital, Kirkenes, Norway; Department of Tumor Biology, Oslo University Hospital, Oslo, Norway; Department of Gastroenterological Surgery, Oslo University Hospital, Oslo, Norway; Division of Cancer Medicine, Surgery and Transplantation, Oslo University Hospital, Oslo, Norway; Institute of Health and Society, University of Oslo, Oslo, Norway; Division of Emergencies and Critical Care, Oslo University Hospital, Oslo, Norway; Department of Health Management and Health Economics, Institute for Health and Society, University of Oslo, Oslo, Norway; Oslo Centre for Biostatistics and Epidemiology, Research Support Services, Oslo University Hospital, Oslo, Norway

**Keywords:** Laparoscopic liver resection, Open liver resection, Parenchyma sparing liver resection, Colorectal liver metastases, Randomized controlled trial

## Abstract

**Background:**

Laparoscopic liver resection is used in specialized centers all over the world. However, laparoscopic liver resection has never been compared with open liver resection in a prospective, randomized trial.

**Methods/Design:**

The Oslo-CoMet Study is a randomized trial into laparoscopic versus open liver resection for the surgical management of hepatic colorectal metastases. The primary outcome is 30-day perioperative morbidity. Secondary outcomes include 5-year survival (overall, disease-free and recurrence-free), resection margins, recurrence pattern, postoperative pain, health-related quality of life, and evaluation of the inflammatory response. A cost-utility analysis of replacing open surgery with laparoscopic surgery will also be performed. The study includes all resections for colorectal liver metastases, except formal hemihepatectomies, resections where reconstruction of vessels/bile ducts is necessary and resections that need to be combined with ablation. All patients will participate in an enhanced recovery after surgery program. A biobank of liver and tumor tissue will be established and molecular analysis will be performed.

**Discussion:**

After 35 months of recruitment, 200 patients have been included in the trial. Molecular and immunology data are being analyzed. Results for primary and secondary outcome measures will be presented following the conclusion of the study (late 2015). The Oslo-CoMet Study will provide the first level 1 evidence on the benefits of laparoscopic liver resection for colorectal liver metastases.

**Trial registration:**

The trial was registered in ClinicalTrals.gov (NCT01516710) on 19 January 2012.

## Background

Colorectal cancer is the third most common cancer worldwide [1]. In 2008 it accounted for 608,700 deaths globally, most of whom died of metastatic tumors [1]. Surgical resection is currently considered the only curative treatment for colorectal liver metastases, with 5-year survival rates following resection between 30% and 58% [2-5].

There are two surgical approaches to resection of liver tumors - open and laparoscopic. No randomized controlled trial comparing the two methods has been completed, but cohort and case-matched studies found a significantly reduced length of hospital stay and need for postoperative opiates after laparoscopic liver resection [6-8]. Long-term oncologic outcomes for laparoscopic liver resection are comparable to open liver resection in retrospective studies [9-11], and meta-analyses of observational studies support these findings [12-14].

Postoperative morbidity after surgery is a major cause of patient suffering and societal costs. The incidence of postoperative morbidity in observational studies on patients undergoing open liver surgery varies from 22% to 47% [15-17], whereas for laparoscopic resection the morbidity rate varies from 11% to 15% [18-20]. Laparoscopic liver resection in patients who previously have undergone open liver resection has a morbidity rate of 29% [21].

In October 2014, the 2^nd^ International Consensus Conference on Laparoscopic Liver Resection was held in Morioka, Japan [22]. At this consensus conference, laparoscopic liver resection was still judged to be in the assessment phase (Balliol grade 3 [23]), as the all the existing evidence is of low quality. The jury recommended that higher quality studies should be performed, using standardized reporting of 90 days mortality and complication rates (article in press, *Annals of Surgery*).

The aim of this randomized controlled trial will thus be to determine whether laparoscopic liver resection of colorectal liver metastases results in less postoperative morbidity than open liver resection.

## Methods/Design

### Funding and ethics approval

The South-Eastern Norway Regional Health Authority funds this study. Approval was obtained from the Regional Committee for Health and Research Ethics (2011/1285/REK Sør-Øst B) and from the Data Protection Official for Research at Oslo University Hospital. The study was registered in Clinicaltrials.gov (NCT01516710) before recruitment started. The guidelines of Consolidated Standards of Reporting Trials (CONSORT) statement will be followed [24].

### Study design

The Oslo-CoMet study is a prospective superiority study. The study is powered to determine whether laparoscopic liver resection of colorectal liver metastases leads to less postoperative morbidity than open liver resection. In addition, the study will also compare epidural patient-controlled analgesia to intravenous patient-controlled analgesia in open liver resection. Patients will be treated equally in every way except the intervention. All patients will undergo an enhanced recovery after surgery (ERAS) program.

All patients will undergo a standardized radiological workup before inclusion, consisting of contrast-enhanced X-ray computed tomography (CT) scans of the thorax and abdomen, and magnetic resonance imaging of the liver (enhanced with liver-specific contrast agent and diffusion-weighted sequences). In cases where extrahepatic metastases are suspected, fludeoxyglucose positron emission tomography scans will be performed. In cases of diagnostic uncertainty, contrast-enhanced ultrasound will be used to increase diagnostic certainty.

### Inclusion and exclusion criteria

The study will include all resections for colorectal liver metastases, except formal hemihepatectomies, resections where reconstruction of vessels/bile ducts is necessary and resections that need to be combined with ablation. At Oslo University Hospital, parenchyma sparing liver resection is an essential part of the multimodal treatment of liver metastases, and therefore fewer formal hemihepatectomies are performed in our institution than in many other institutions. Patients must be willing and able to give informed consent. Exclusions include patients with previous liver ablations, patients where resection of primary tumor is planned for the same procedure and patients with nonresectable extrahepatic disease.

### Primary and secondary endpoints

The primary endpoint of the Oslo-CoMet study is the rate of postoperative morbidity and mortality. Morbidity will be registered using the Accordion-classification and the Comprehensive Complication Index during hospital stay, at discharge and at the outpatient clinic after 30 days [25-27].

**Figure 1:**
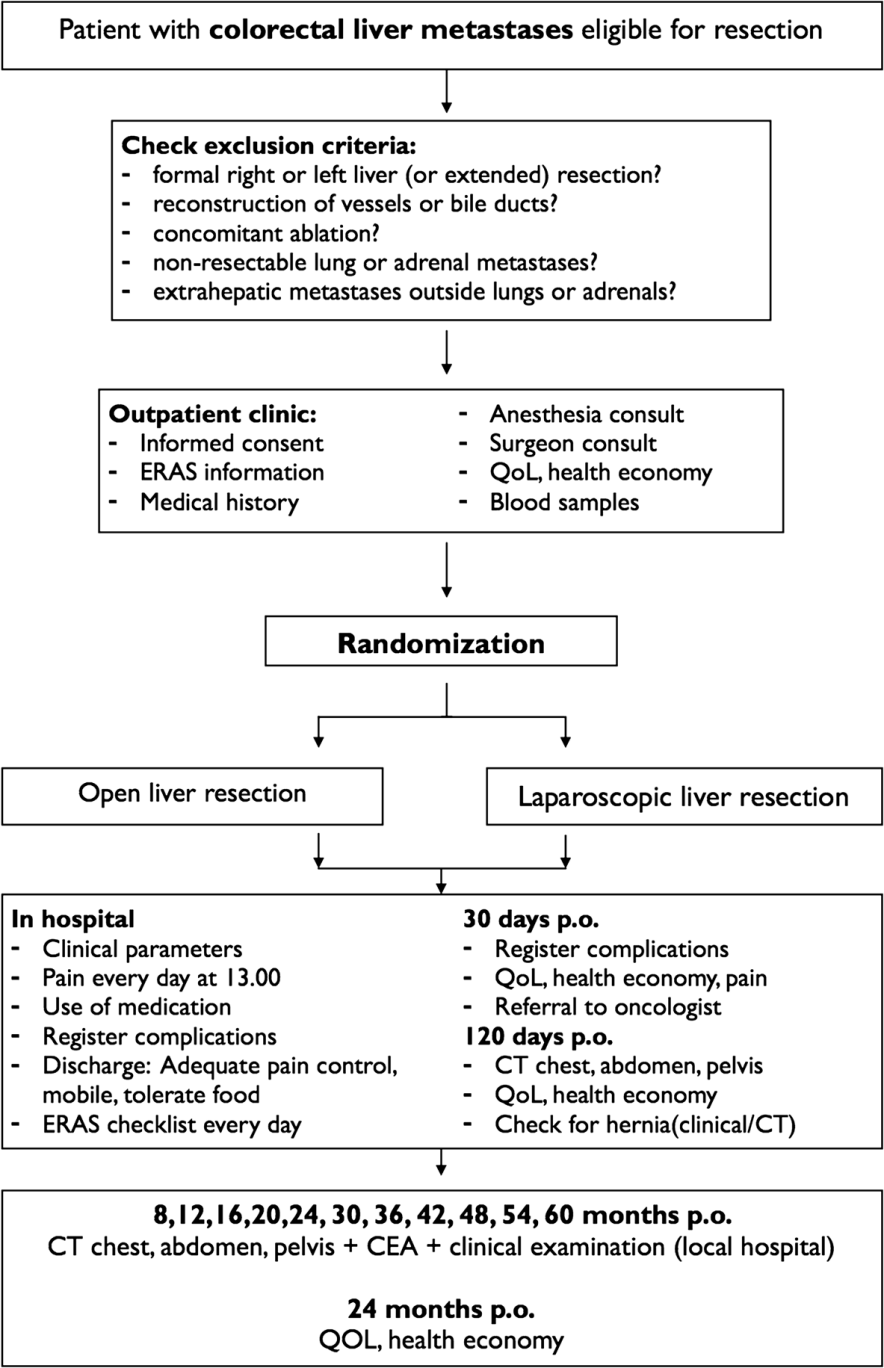
**Oslo-CoMet study flowchart.** CEA, carcinoembryonic antigen; CT, computed tomography; ERAS, Enhanced Recovery After Surgery; p.o., postoperative; QoL, quality of life.

Secondary endpoints include 5-year overall, disease-free and recurrence-free survival, recurrence pattern and management of recurrence. Differences in the inflammatory response, resection margins, hospital and societal costs, health-related quality of life, hernia development, postoperative pain, and intraoperative unfavorable incidents (according to the modified Satava classification [28,29]). A biobank with blood and tissue from tumor and healthy liver will be established for molecular analyses (Figure 1).

### Management of postoperative pain

The optimal management of pain following major abdominal surgery is subject to on-going debate. Epidural analgesia after open abdominal surgery has been shown to reduce pain [30], postoperative ileus [31] and inflammatory response [32,33], but it also carries rare but severe complications such as post-dural puncture headache and paralysis of lower extremities. A recent systematic review concludes that large-scale prospective randomized trials are required to find the optimal management of postoperative pain after open liver resection [34]. In our institution, postoperative epidural analgesia is used for open liver resection, but not for laparoscopic resection. Therefore, we decided to randomize between epidural analgesia and intravenous opioids in the open surgery group.

All patients will receive anesthesia and postoperative pain management guided by the same standardized protocol. The area of planned incision (either open or trocar) will be infiltrated by local anesthesia. At the start of the operation, 8 mg intravenous dexamethasone will be administered, while intravenous paracetamol and ketorolac will be administered at the end of anesthesia. Patients in whom an epidural catheter is placed will not receive ketorolac because of the risk of epidural hematoma. On the day of surgery and on postoperative days 1 and 2, the patient will receive paracetamol 1 g 6-hourly and intravenous ketorolac 30 mg 8-hourly. Following this, the patient will receive oral paracetamol 1 g 6-hourly and oral diclofenac 50 mg 8-hourly. In the laparoscopic group, and in the part of the open group not randomized to receive epidural analgesia, a patient-controlled analgesic pump (PCA) will deliver intravenous opioid pain relief (ketobemidone) in bolus doses of 0.1 mg/kg body weight. According to unit policy, the PCA will be discontinued as soon as possible, and patients will then be offered oral diclofenac, supplemented by paracetamol and intravenous or oral opioids.

### Enhanced recovery after surgery

The ERAS program is now an established part of perioperative care in many centers, and has been adapted for liver surgery [35,36]. In addition to reducing the length of stay after open liver resection [35], ERAS provides a framework for standardizing the perioperative management of both treatment groups in our trial, reducing bias. If a patient will need intensive care treatment, or for other reasons is unable to follow the ERAS program, it will be initiated as soon as the patient is well enough.

### Randomization procedure

At the outpatient clinic, patients will receive both verbal and written information about the study. When patients have given their written, informed consent, randomization will be performed. Randomization will be generated in proportion 2:1:1 (laparoscopic resection with intravenous analgesia: open resection with epidural analgesia: open resection with intravenous analgesia). The software used for registration of patient data will generate randomization, the details of which are available on request. The patient will be informed about the result of randomization upon admission to Oslo University Hospital on the day before surgery.

### Blinding

A blinded assessor will evaluate the primary endpoints separately using the nurses’ electronic patient record system.

### Confidentiality, data handling and monitoring

Every patient will be assigned a unique, encoded number. A designated information technology manager will control the decoding key. Trial data will be stored on a secure server with regular backup, and all activity on the server will be traced. Patients can withdraw from the study at any time without consequences, and data from these patients will be deleted. A Data and Safety Management Board (DSMB) will be established [37]. The DSMB consists of a chairperson, a medical specialist and an independent statistician, and the board will assess safety issues when necessary.

### Surgical technique

Surgical technique will be at the discretion of the operating surgeon. For open surgery, an L-shaped, subcostal or midline incision will be used according to tumor size and location. For laparoscopy, three ports are used initially, with addition of extra ports or, in selected cases, hand ports as necessary.

For both open and laparoscopic surgery, parenchyma will be divided with electrosurgical instruments, mainly LigaSure® (Covidien, Mansfield, MA, USA), Thunderbeat® (Olympus, Tokyo, Japan) or Cayman® (B.Braun, Melsungen, Germany), sometimes assisted by ultrasonic aspirators, mainly SonoSurg aspirator® (Olympus, Tokyo, Japan) and Söring aspirator® (Söring, Quickborn, Germany). Endoscopic staplers, Endo-GIA® (Covidien) and Endopath® (Ethicon, Bridgewater, NJ, USA), will be used for dividing large vessels and sometimes also for parenchyma division. When the Ligasure® is not used for this purpose, the liver capsula will be divided with ultrasonic scissors, such as Sonicision® (Covidien), SonoSurg scissors® (Olympus) or Harmonic scalpel® (Ethicon).

### Inflammatory response

The inflammatory response will be evaluated in the first 45 patients included in the study by measuring selected alarmins, cytokines, chemokines and terminal complement complex in fresh frozen ethylenediaminetetraacetic acid plasma taken before surgery, every hour during surgery and at 2, 6 and 24 hours after surgery. Due to financial limitations, it will not possible to perform this comprehensive evaluation of the inflammatory response on all patients in the study. Based on previous experiences [38], 45 patients are considered to be sufficient to show differences in the inflammatory response after open and laparoscopic liver resection.

### Resection margins

The operating surgeon will assess macroscopic resection margins without slicing the specimen. Before surgery, each tumor will be given a number, the largest being number 1 and so on, and the resection margin will be evaluated for every resected tumor. The final microscopic margin will be measured during routine histological assessment by the histopathologist.

### Hospital and societal costs

Costs for both open and laparoscopic surgery will be estimated alongside the clinical trial at the individual patient level, using a health care and a societal perspective. For the initial hospital stay, both direct patient-related activities and indirect nonpatient-related costs (overhead costs) will be estimated. To estimate the exact resource use in the operating theatre, we will quantify the personnel resources and the disposable and nondisposable equipment used during surgery in a subpopulation of 50 patients. Cost drivers (complications) will be quantified by the Accordion system [25]. Health care and societal costs after discharge from Oslo University Hospital will be assessed by questionnaires that the patients fill in at the 30-day and 4-month follow-up. For resource use after the 4-month follow-up, the Oslo-CoMet study will be linked to national registers with data on resource use in the health care sector at the individual patient level. The registers include information on the use of specialist health care (the Norwegian Patient Registry), primary care services and services from privately practicing physicians, specialists and psychologists (the KUHR-registry), the use of care services (the IPLOS-registry) and dispensed prescription drugs (the Norwegian Prescription Database).

### Health-related quality of life

To assess patient health-related quality of life, patients will fill in the short-form 36-item (SF-36) version 2.0 [39] at baseline, and at the 30-day and 4-month follow-up. After 24 months, the SF-36 will be mailed to patients with encouragement to return it to Oslo University Hospital by mail. The SF-36 includes one multi-item scale measuring several health domains: (1) physical functioning, (2) role limitations caused by physical health problems, (3) bodily pain, (4) general health, (5) vitality, (6) social functioning, (7) role limitation caused by emotional problems, and (8) mental health. Scores per dimension range from 0 to 100; higher scores indicate better health status. SF-36 has been translated to several languages, including Norwegian. It has been tested for psychometric properties in several countries, including Norway, with internal consistency (Cronbach’s alpha) ranging from 0.80 (role emotional) to 0.93 (bodily pain) [40]. The SF-36 has previously been used to show differences in health-related quality of life for several surgical procedures, including laparoscopic and open donor nephrectomy [41]. The SF-36 will also be transformed into the SF-6D, which is a preference-based measure of health, also referred to as utilities. This is done by running an algorithm on 6 out of the 36 dimensions in the SF-36. The algorithm is based on a population-based study in the UK [42]. Utilities are measured on a 0 (dead) to 1 (best possible health) scale [43], and will, in combination with the estimated costs of the two procedures, be used in the cost-utility analysis in the Oslo-CoMet study. The disease specific EORTC QLQ-30 LMC-21 [44] will be used in a subgroup of the study patients in order to make a comparison between the two forms. This has previously never been performed on patients undergoing liver resection. LMC-21 will be collected at the same time points as SF-36.

### Hernia development

The incision type and length, and the port size and number will be recorded. The incidence of incisional hernias will be evaluated during clinical examination at the outpatient clinic after 4 months, and using the abdominal CT taken at oncological follow-up every 4 months thereafter.

### Postoperative pain

Postoperative pain will be recorded when the patient arrives at the ward from the postoperative care department, and at 14.00 on postoperative days 1 to 5. Trained ward nurses will perform pain registration using the validated, verbally administered 11-point Numeric Rating Scale (0–10). If the patient is fully mobilized and discharged before day 5, patients will perform the pain registration themselves after receiving careful instructions from nurses. The Numeric Rating Scale is closely correlated to the Visual Analogue Scale [45,46], and can be easier to complete for postoperative patients as it can be administered verbally.

### Follow-up

Thirty days after surgery, participants in the Oslo-CoMet study will meet a surgeon in the outpatient clinic for inspection of wounds, registration of any complications arising after discharge, review of histology and planning of adjuvant chemotherapy. Patients will also fill in the SF-36 and a questionnaire recording resource use at the 30-day follow-up, at 4 months and 2 years. Four months after surgery, and every 4 months for the first 2 years, patients are examined with CT scans of the thorax and abdomen, measurement of carcinoembryonic antigen and clinical examination by a surgeon. After 2 years, the surveillance interval will be every 6 months until at least 5 years or dropout. The follow-up is identical to Norwegian guidelines.

### Biobank

In this prospective randomized trial there will also be potential for posing questions of more basic and translational nature and, for this purpose, a comprehensive biobank will be generated. All specimens are transported on ice to the pathologist directly after extraction. The pathologist will immediately collect tissue from both healthy liver and resected tumor. This will be done carefully so routine evaluation of resection margins not will be compromised. Tissue will be snap frozen in liquid nitrogen and placed in an ultrafreezer (−80°C).

A systems biology approach will be taken, and from primary tumors, normal liver tissue and blood derivatives (mononuclear cells, plasma and serum), DNA, RNA and protein extracts will be generated. A range of molecular analyses will be performed using high throughput array-based strategies for analysis of, for example, genomic variation, gene expression, protein modifications and metabolic profiles. Furthermore, formalin-fixed, paraffin-embedded tumor tissue from routine pathology processing will be available, and will be collected from referring hospitals for the primary tumors to make possible the generation of tissue microarrays for comparing characteristics of the metastatic lesions with the primary tumors by, for instance, immunohistochemistry. Results from all these analyses will be correlated to disease outcome, but also with other study endpoints; for all included patients, a comprehensive set of clinical information will be available, allowing the investigation of prognostic and predictive biomarkers.

### Video storage

In order to extend the use of laparoscopic video, an application for streaming to a repository will be used. The repository is hosted on a server in the hospital enterprise network, together with a database of the videos. The objective is to provide a streamlined platform to process surgical videos for clinical quality control and educational purposes. The video streaming application allows real-time tagging of events during surgery, postprocedure tagging and video editing to produce a short video of highlights. A set of predefined tagging parameters for laparoscopic liver metastasis surgery is used. The laparoscopy videos will be exported to the main data collection platform of the project.

### Database

**Figure 2:**
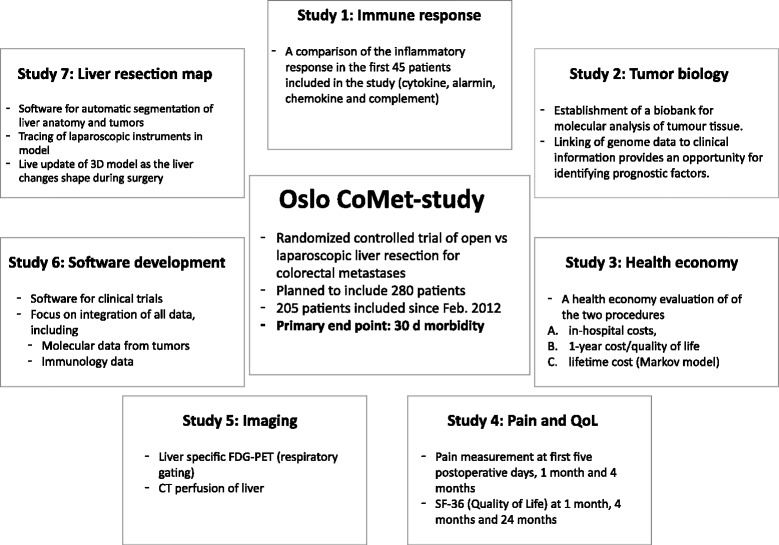
**Oslo-CoMet study: overview of substudies and translational research.** SF-36, Short Form 36; FDG-PET, fludeoxyglucose positron emission tomography; CT, Computed Tomography.

The case report form (CRF) for each patient in the study will be implemented as an XML document which will be stored in a database. When changes are made on a CRF, a copy of the old CRF will be stored together with a log showing who made the changes. Users will not have access to the CRF log. For security reasons there will be one main copy of the database with full patient identification, and two copies with modifications: one anonymized database, and one with de-identified information. Analysis will never be performed on the main database (Figure 2).

### Registry

A registry will be created of all patients who give consent to participate but for some reason cannot be randomized. A CONSORT flow diagram will be created showing the total number of patients that were operated for colorectal liver metastases in the study period, and the reasons for all exclusions of patients that were eligible for the study.

### Statistics

After reviewing our own data and the literature, we expect a 13% morbidity rate in the laparoscopic group [19,47] and a 27% rate in the open group [17,19,48]. With a significance level of 5% and two-sided test, 254 patients will be needed to complete the study with 80% power. To allow for 10% dropout, we plan to include 280 patients.

Descriptive statistics will be used to describe baseline data. We will use statistical methods for contingency tables and categorical data to analyze rates and proportions at single time points. Statistical analyses for repeated measures will be used to investigate developments over time. To assess mortality, survival analysis will be performed.

### Intention-to-treat

Analysis of all patients will be performed according to the intention-to-treat principle. However, data will also be presented per-protocol and for the actual treatment groups. This especially concerns converted laparoscopic operations, where patients will cross over to the open group. We consider it important to present also the actual treatment data, as they will be useful especially for systematic reviewers.

## Discussion

### Justification of the trial

Currently only low-level evidence supports the implementation of laparoscopic liver resection over open liver resection for the surgical management of hepatic colorectal metastases. Thus, the trial is scientifically and ethically justified.

### Selection of endpoints

The primary endpoint of this study is complications within 30 days, reported using the Accordion system [25] and the Comprehensive Complication Index [27]. Serious complications after surgery have been shown to influence survival for several surgical treatments, including liver resection for colorectal metastases [17], gastric resection for cancer [49] and pancreatoduodenectomy for periampullary cancer [50]. However, complication data on atypical liver resections are not easily available, as most reported material on open liver resections includes formal and extended resections as well as atypical resections. Thus a moderate 27% expected complication rate for open resections [17,19,48] and 13% for laparoscopic resections was chosen [19,47,51,52]. In the years prior to the study, most atypical liver resections in our hospital were performed by laparoscopy, and currently more than 600 laparoscopic major and minor liver resections have been performed at Oslo University Hospital.

### Enhanced recovery after surgery

The optimization of perioperative care has received much attention over the last two decades. ERAS protocols aim to attenuate stress response, thus reducing complications, length of stay and improving patient comfort. An ERAS protocol, which has strict guidelines for oral intake, use of analgesics and early mobilization, ensures similar treatment of both groups in a surgical randomized controlled trial. This will improve the trial quality not only by enhancing recovery in both groups, but by improving the performance of the control group. The Orange II trial also follows an ERAS protocol [36].

### Single center versus multicenter

The Oslo-CoMet study is a single center study from a high volume Hepato Pancreato Biliary (HPB) laparoscopy center. After the merger of hospitals in Oslo, our HPB centre has solitary treatment responsibility for the 2.8 million people in the South-Eastern Norway Regional Health Authority [53]. This allowed to us create one algorithm for radiology, recruitment, surgical technique, chemotherapy and follow-up. These advantages, together with the lack of possible partners (there are no comparable laparoscopic HPB centers in the Nordic countries), helped the study group decide that a single center study was most suitable.

The Intervention Centre at Oslo University Hospital is a research and development unit with long experience in development and implementation of surgical procedures. The centre has a framework well suited for running a randomized controlled trial. The study is a joint venture between the Department of HPB Surgery, Oslo University Hospital, and the Intervention Centre, with direct funding from the South-Eastern Norway Regional Health Authority.

### Choice of resections

The study will include all liver resections less than three segments, atypical and anatomical. Thus, there will be a great diversity of operations included, from small wedge resections to large parenchyma sparing resections. The randomization is expected to equalize these differences. This design closely reflects the reality of todays parenchyma sparing treatment of colorectal liver metastases [54,55]. The Orange II plus trial, a multicenter trial of laparoscopic versus open hemihepatectomy, is currently recruiting patients and will supplement the scientific picture with results of formal hepatectomies. The Oslo-CoMet trial will also verify the safety and effect of parenchyma sparing liver resections for colorectal liver metastases.

### Blinding

Blinding of patients for a surgical procedure can be performed using large wound dressings, but the epidural analgesia will be almost impossible to hide. We found that adequate blinding of surgeons and nursing personnel would be virtually impossible in our department. The reason for blinding of patients is primarily to avoid the change of behavior in the trial [56]. As the patients are informed about randomization only the evening before surgery, we judge that possibility for change of behavior is small. The primary endpoint is morbidity, an objective parameter that the patient cannot influence. We admit, though, that the reporting of pain, quality of life and to a certain degree the length of stay could be influenced by the fact that the patients know which operation they have had.

We still realize that lack of blinding may weaken the study. Patients receiving their preferred treatment may perform better than they otherwise would have, and *vice versa*. Our experience is, however, that some patients will prefer the “safe” option of open surgery while others will prefer the “modern” laparoscopic surgery.

## Trial status

As of 10 January 2015, 200 patients have been included in the Oslo-CoMet study. The samples for the first immunology study have been analyzed and results will be published shortly. The first quality of life data will be published when 4 months follow-up of the first 120 patients is ready. Tissue from the bio bank is being processed and more than 50 samples have been analyzed using the Ion Torrent® platform (Life Technologies, Grand Island, NY, USA). Data concerning primary and secondary endpoints will not be published until the expected number of patients is reached. No interim analysis is planned but safety issues are continuously evaluated.

The Oslo-CoMet study (NCT01516710) will to our knowledge be the largest randomized controlled trial on laparoscopic versus open resection of colorectal liver metastases. The Orange II (NCT00874224) and Orange II plus (NCT01441856) trials are currently recruiting. A Chinese (NCT01768741) and a South Korean (NCT00606385) trial of hepatocellular carcinoma resections are also recruiting patients. Together these trials will provide level 1 evidence on the comparison of open and laparoscopic liver surgery. The Oslo-CoMet study is planned to complete recruitment in 2015.

## Abbreviations

CONSORT: Consolidated Standards of Reporting Trials
CRF: case report form
CT: computed tomography
DSMB: Data and Safety Management Board
ERAS: enhanced recovery after surgery
HPB: Hepato Pancreato Biliary
PCA: patient-controlled analgesic pump
SF-36: short-form 36-item
